# Usability of German hospital administrative claims data for healthcare research: General assessment and use case of multiple myeloma in Munich university hospital in 2015–2017

**DOI:** 10.1371/journal.pone.0271754

**Published:** 2022-07-28

**Authors:** Amal AlZahmi, Irena Cenzer, Ulrich Mansmann, Helmut Ostermann, Sebastian Theurich, Tobias Schleinkofer, Karin Berger

**Affiliations:** 1 Department of Medicine III, Ludwig Maximilians University Hospital, Munich, Germany; 2 Division of Geriatrics, Department of Medicine, University of California, San Francisco, San Francisco, CA, United States of America; 3 Institute for Medical Information Processing, Biometry, and Epidemiology–IBE, Ludwig Maximilians University, Munich, Germany; 4 Faculty of Medicine, DIFUTURE Data Integration Center of Ludwig Maximilians University Hospital, Munich, Germany; 5 University Hospital, LMU Munich, Munich, Germany; 6 Cancer- and Immunometabolism Research Group, Ludwig Maximilians University Hospital, Gene Center, Munich, Germany; 7 German Cancer Consortium (DKTK), Munich Site, and German Cancer Research Center, Heidelberg, German; URCEco Ile de France Hopital de l’Hotel Dieu, FRANCE

## Abstract

**Objectives:**

To assess the usability of German hospital administrative claims data (GHACD) to determine inpatient management patterns, healthcare resource utilization, and quality-of-care in patients with multiple myeloma (PwMM).

**Methods:**

Based on German tertiary hospital’s claims data (2015–2017), PwMM aged >18 years were included if they had an International Classification of Diseases, Tenth Revision, code of C90.0 or received anti-MM therapy. Subgroup analysis was performed on stem cell transplantation (SCT) patients.

**Results:**

Of 230 PwMM, 59.1% were men; 56.1% were aged ≥65 years. Hypertension and infections were present in 50% and 67.0%, respectively. Seventy percent of PwMM received combination therapy. Innovative drugs such as bortezomib and lenalidomide were given to 36.1% and 10.9% of the patients, respectively. Mean number of admissions and mean hospitalization length/patient were 3.69 (standard deviation (SD) 2.71 (1–16)) and 12.52 (SD 9.55 (1–68.5)) days, respectively. In-hospital mortality was recorded in 12.2%. Seventy-two percent of SCT patients (n = 88) were aged ≤65 years, 22.7% required second transplantation, and 89.8% received platelet transfusion at a mean of 1.42(SD 0.63 (1–3)).

**Conclusion:**

GHACD provided relevant information essential for healthcare studies about PwMM from routine care settings. Data fundamental for quality-of-care assessment were also captured.

## Introduction

Secondary data sources have been increasingly used in health services research over the past few years [1–3]. Administrative medical data, also known as “claims data” are an example of secondary data collected for purposes other than scientific research [4]. The literature describes secondary databases as appropriate to answering healthcare questions from different perspectives such as healthcare providers and payers [5, 6]. Secondary databases also reflect upon healthcare utilization and treatment patterns in routine care settings [5–7]. They have been extensively used to address concerns related to, e.g., epidemiology, drug safety and effectiveness, treatment patterns, and impact of healthcare policies and quality of care assessment [8–12]. They include large study populations and special subpopulations that are difficult to recruit for prospective observational studies, rendering them as potential convenient alternatives for health research studies [5].

In Germany, different administrative claims data sources are available for research e.g. hospital-based administrative claims data like the data collected in the format of §21 data set [13], statutory claims data, data from the office-based physicians association and data from federal databases [5, 9, 14–17]. All databases differ slightly from each other in terms of the data granularity and extent of data variables they contain and at certain points these databases might be complementary. The use of claims data in health services research has increased in Germany over the past decade [5, 18]. However, most of such studies have involved claims data from German statutory health insurance (SHI) databases [8, 15, 16]. Despite the fact that single-payer and SHI databases include a wide range of information important for health services studies from cross-sector outpatients and emergency departments visits, they are limited on variables required for quality-of-care assessment from inpatient all-payer aspects [19]. On the other hand, hospital administrative claims data provide more information about all therapeutics and diagnostic interventions that are reimbursed beyond the DRG system from inpatient perspectives irrespective of their health insurance. Thus, they could serve as a better alternative for quality-of-care assessment and benchmark evaluation studies from inpatient all-payer perspectives. Moreover, when combined to other data; e.g., SHI claims data, and patient medical record; they could provide a comprehensive evaluation of any predefined disease condition from real-world settings.

For rare conditions such as multiple myeloma (MM), secondary databases can serve as ideal sources for evidence concerning management patterns and healthcare resource utilization from routine care settings. MM is an incurable disease of plasma cells primarily affecting older people [1, 3, 20, 21]. In Germany, MM is the third most common hematologic neoplasm after leukemia and non-Hodgkin lymphoma [22]. The treatment landscape for MM is continuously changing over time, although it is primarily directed at providing symptomatic relief, controlling the disease, and increasing the overall survival of patients [1, 23–25]. Despite the improvement in the overall survival of patients with MM using novel agents, MM poses an economic burden that must be evaluated and addressed within routine care settings [2]. Previous studies on MM in Germany conducted using real-world data were based primarily on surveys [26, 27] and patient charts [28]. Depending on the quality and granularity of German hospital databases, they may provide valuable information for epidemiologic and health services studies, benchmark evaluations, and quality of care assessments. To the best of our knowledge, there is yet no study in Germany on the use of hospital administrative claims databases in health services research to address MM-related issues.

This study was conducted to assess the usability of German hospital administrative claims data to determine inpatient management patterns, healthcare resource utilization, and quality-of-care. As a use-case we referred on patients suffering from MM.

## Methods and materials

### Study design and data source

All analyses were conducted on the basis of hospital administrative claims data of the Ludwig Maximilian University hospital, a tertiary university hospital with a specialized hematology–oncology department containing a specialized center to treat MM cases.

Claims data in German hospitals are collected using a uniform structure of §21 dataset [13], which is a performance and flat-rate dataset based on the German diagnosis-related group (G-DRG) system and the International Classification of Diseases, Tenth Revision, German Modification (ICD-10-GM) system. It contains information on all health services (e.g., diagnostic and therapeutic procedures reimbursed beyond the DRG system) provided to patients during their hospitalization irrespective of their health insurance. We obtained hospital administrative claims data based on §21 dataset structure from the KUM for the period 2015–2017. The dataset contains information on patients’ identifiers, case number, pay area (e.g., DRG, additional fees, fees for novel interventions), health insurance ID, demographics (age, gender), reason for admission (primary diagnosis vs. secondary diagnosis), admitting department, diagnosis code, localization of diagnosis, procedure codes, date of procedure, admission and discharge dates and reason for discharge/transfer. The hospital administrative claims dataset was anonymized by the Trust Center and processed by the Medical Data Integration Center, both of which are located at Ludwig Maximilians University (LMU) and KUM inside the Data Integration for Future Medicine (DIFUTURE) consortia of the Medical Informatics Initiative (MII) that is funded by the German Federal Ministry of Education and Research (BMBF). In this context, the hospital administrative claims dataset was used for MII’s national, cross-consortia demonstrator study after obtaining approval from the Ethical Review Board of LMU’s Faculty of Medicine and KUM’s Data Protection Officer. We followed the RECORD checklist to construct this manuscript [29].

#### Inclusion criteria

The study sample composed of patients with multiple myeloma with inpatient records during the period of 2015–2017. Patients aged >18 years were included if they fulfilled at least one of the following conditions: (1) at least one inpatient MM diagnosis (ICD-10 = C90.00 and C90.01) as the primary reason for hospitalization or (2) received anti-MM therapy. The ICD-10 code for identifying patients with MM was validated elsewhere [30].

### Outcome measures

We began by evaluating data availability in the hospital administrative claims dataset. To compile a list of necessary data elements, we performed a narrative literature review of papers that investigated MM using administrative claims data. We evaluated research questions, methods, data required to answer each research question, and prominent findings in the identified reports. Finally, we evaluated the presence of each one of these elements in the hospital administrative claims dataset. We used a list of the required procedure codes (OPS-codes) and specific ICD-10 codes to identify medications used, procedures performed, and diseases diagnosed (S1 Table). This list should represent almost a complete variable list required to answer healthcare research questions. It should also serve as a blueprint for future studies aiming at linking multiple secondary data sources by providing the sources for each data variable. We used this list to identify the extent of data variables present in our dataset.

After identifying data elements present in the hospital administrative claims dataset, we conducted a specific analysis to evaluate the comprehensiveness and usability of such data elements. First, we examined the demographic characteristics of patients with MM, including age and sex. Second, we examined their clinical characteristics in terms of disease stage and severity, comorbid conditions, disease- and/or treatment-related complications, and in-hospital mortality. Third, we examined management patterns in terms of prescribed medications, line of therapy, and therapeutic and diagnostic procedures. Anti-MM therapy included administration of bortezomib (OPS-code = 6–001.9), lenalidomide (OPS-code = 6-003.g), or combination therapy (OPS-codes = 8–542, 8–543, and 8–544). Finally, healthcare utilization in terms of health resources consumption was defined as the number of readmissions that lasted >24 h, length of hospitalization, and therapeutic and diagnostic procedures performed.

We conducted a subgroup analysis on SCT patients because it served as a homogeneous subgroup of patients with MM, and an index date from the start of the procedure could be set. We assessed the possibility of evaluating these patients’ clinical characteristics in terms of complications after SCT, management pattern in terms of treatment received, and reason for hospitalization after the procedure.

### Statistical analysis

Categorical variables were presented descriptively as counts and percentages. Continuous variables were presented as mean and standard deviation (SD). Statistical analyses were conducted using the SAS 9.4 software (X64 10HOME platform, Copyright (c) 2002–2012 by SAS Institute Inc., Cary, NC, USA). A sunburst chart was produced using Rstudio 3.6.1 (Version 1.2.500^©^ 2009–2019, Inc.).

## Results

The hospital administrative claims dataset contained variables required for case identification and evaluation of age and sex distribution among patients with MM (Table 1). It included some but not all information required to evaluate patients’ clinical characteristics. It contained variables required for identifying possible comorbid conditions and disease- and/or treatment-related complications based on ICD-10 codes. It also included information for identifying in-hospital mortality under a variable termed “discharge/transfer reason.” The diagnosis date was not recorded in the dataset thus we could not maintain the same follow-up period for all the patients. It also limited our ability to rigorously evaluate the chronological sequence of events and distinguish between unrelated comorbid conditions, and disease- and/or treatment-related complications. In other words, we could not set an index date from disease onset and follow up patients’ clinical history over time to identify the occurrence and development of other conditions or complications. Moreover, details regarding laboratory and radiological findings and disease stage and severity were unavailable, hindering the evaluation of disease stage and severity as well as disease risk assessment.

**Table 1:** Data completeness evaluation tool.

Outcome measures	Question	Data availability	
		Yes	No
**Demographics**	Can the patients be identified using the dataset?	X	
	What is the age distribution of the identified group of patients?	X	
	What is the sex distribution of the disease group?	X	
**Clinical characteristics**	Can the diagnosis of multiple myeloma be confirmed using the dataset?		X
	Can the disease stage of patients with multiple myeloma be assessed using the dataset?		X
	Can the disease risk in the identified group of patients be assessed using the dataset?		X
	Which comorbid conditions were documented in the identified multiple myeloma group?	X	
	Which disease- and/or treatment-related complications were documented in the identified multiple myeloma group?	X	
	Was in-hospital mortality of patients with multiple myeloma documented in the dataset?	X	
**Management**	What medications are used for: • Front-line therapy? • Relapsed/refractory multiple myeloma? • Consolidation therapy? • Maintenance therapy? • Supportive care?		X
	What therapeutic procedures were performed on the identified patients? • Therapeutic plasmapheresis • Hemodialysis • Blood transfusion • Thrombocyte transfusion • Stem cell transplantation	X	
	Which diagnostic procedures were performed on the identified patients? • Computed tomography (CT) • Magnetic resonance imaging (MRI) • Positron emission tomography (PET)/CT • Conventional radiographs • Immunocytochemical detection of circulating tumor cells • Bone marrow biopsy • Genetic testing • Pulmonary function test • Endoscopy	X	
**Health resource utilization**	How frequent were patients with multiple myeloma admitted to the hospital?	X	
	How long did patients with multiple myeloma stay in the hospital?	X	
	Which health services are used by the identified group of patients? • Laboratory: (Complete blood count, serum/urine protein electrophoresis, cytogenetic test, bone marrow aspiration/biopsy) • Radiology: (CT, MRI, PET-CT) • Therapeutic: (chemotherapy, radiotherapy, immunotherapy, stem cell transplantation, blood transfusion, thrombocyte transfusion, plasmapheresis, dialysis, antiviral, antifungal, antibiotic and supportive therapy*)	Not complete	

Abbreviations

CT: Computed tomography

MRI: Magnetic resonance imaging

(PET)/CT: Positron emission tomography

For evaluating management patterns, we could identify prescribed medications and diagnostic and therapeutic procedures performed using pre-specified procedure codes. This approach allowed us to evaluate the treatment provided and the diagnostic and therapeutic procedures performed in terms of documentation frequency. However, because of missing diagnosis date, we could not construct a line of therapy. Treatment initiation date, therapy duration and dose, and evidence of treatment discontinuation/switching were not recorded, limiting the appropriate evaluation of treatment patterns.

The hospital administrative claims dataset contained information for assessing healthcare utilization in terms of the number of hospital admissions and length of hospital stays. However, the dataset was limited to a single hospital department, and no data on outpatient and emergency department visits were available. Therefore, admissions to other departments within the same hospital were not captured in the dataset.

### Description of study sample

We identified 325 patients with a MM diagnosis code, of whom 222 (68.3%) were admitted with MM as the primary reason for admission. An additional eight patients who received anti-MM therapy but were not admitted primarily for MM were included. Overall, 230 patients with MM were included in the study.

Patients’ mean age at first admission was 65 years (SD = 12), and there were 136 (59.1%) men. In total, 196 (85.2%) were readmitted to the same hospital within 1 year (Table 2). Hypertension (50.0%), chronic kidney disease (32.6%), and other tumors (21.7%) were the most documented comorbid conditions (Table 1). Infection (67.0%), neutropenia (50.0%), and thrombocytopenia (50.3%) were the most documented disease- or treatment-related complications. In-hospital mortality was reported in 12% of patients.

**Table 2:** Demographics, clinical characteristics, and management patterns of patients with multiple myeloma.

Patients with MM	N = 230 n (%)
**I. Patient demographics:**	
**a. Sex:**	
Male	136 (59.1%)
Female	94 (40.9%)
**b. Age group**	
<65 years	101 (43.9%)
65–70 years	37 (16.1%)
≥70 years	92 (40.0%)
Mean age at first admission (Standard deviation)	65 (12)
(min–max)	(34–89)
**II. Readmission episodes following first admission/year**	
Within 1 year	196 (85.2%)
Within 2 years	22 (9.6%)
Within 3 years	4 (1.7%)
**III. Comorbid conditions:**	
Hypertension	115 (50.0%)
Congestive heart failure	36 (15.6%)
Cerebrovascular disease	15 (6.5%)
Chronic kidney disease	75 (32.6%)
Other tumors^†^	50 (21.7%)
Diabetes mellitus type 2	36 (15.7%)
Ischemic heart disease	29 (12.6%)
**IV. Disease-related and/or treatment-related complications:**	
Skeletal-related events	76 (33.0%)
Anemia	51 (22.2%)
Drug-induced anemia	89 (38.7%)
Renal complications	44 (19.1%)
Gastrointestinal bleeding	9 (3.9%)
Infections^‡^	154 (67.0%)
Urinary tract infection	51 (22.2%)
Thrombocytopenia	116 (50.3%)
Peripheral neuropathy	26 (11.3%)
Neutropenia	115 (50.0%)
End-stage renal disease	54 (23.5%)
Underweight^§^	7 (3.0%)
**V. In-hospital mortality**	28 (12.2%)
**VI. Management pattern**	
**a. Anti-multiple myeloma therapy**	
Combination therapy	162 (70.4%)
Bortezomib	83 (36.1%)
Lenalidomide	25 (10.9%)
Immune therapy	29 (12.6%)
**b. Supportive therapy**	
Pain medication	14 (6.1%)
Lipegfilgrastim^¶^	36 (15.7%)
Antifungal medications	42 (18.3%)
**c. Therapeutic procedures**	
Stem cell transplantation	88 (38.3%)
Blood product transfusion • Platelet transfusion	164 (71.3%) 117 (50.9%)
Stem cell collection	73 (31.7%)
Hemodialysis	23 (10.0%)
**d. Diagnostic procedures**	
Computed tomography scan	187 (81.3%)
Pulmonary function test	120 (52.2%)
Bone marrow biopsy	93 (40.4%)
Magnetic resonance imaging	62 (27.0%)
Diagnostic endoscopy	31 (13.5%)

^†^Primary benign, malignant, and unspecified tumors as well as secondary tumors were grouped together under one category.

^‡^Infections included cholera, typhoid and paratyphoid, salmonella infections (enteral salmonella, sepsis salmonella, localized salmonella, and unspecified salmonella); shigellosis; bacterial stomach infection (*E*.*coli*); bacterial enteritis; foodborne bacterial illness; amebiasis; intestinal diseases caused by protozoa; viral-induced gastroenteritis; other unspecified infectious gastroenteritis and colitis of unspecified origin; meningococcal infection; streptococcus infections; unspecified sepsis, bacterial infections of unspecified localization; other viral encephalitis not otherwise classified; unspecified viral encephalitis; viral meningitis; other unspecified viral infection of the central nervous system; viral infections of unspecified localization; streptococci and staphylococci as the cause of infections classified in other chapters; other specified bacteria as the cause of diseases classified in other chapters; viruses as the cause of diseases classified in other chapters; other specified infectious agents as the cause of diseases classified in other chapters; herpes simplex infection; varicella infections; herpes zoster infection; smallpox; rubeola; and viral-induced skin and mucosal diseases.

^§^ICD-10 codes for underweight include R63.4 = abnormal weight loss and R64 = cachexia.

^¶^Lipegfilgrastim is a medication used to treat neutropenia in patients with cancer.

Abbreviations

MM: Multiple myeloma

Combination therapy was administered to 70.4% of patients. Bortezomib (36.1%) and lenalidomide (10.9%) were most frequently administered to the patients, whereas 38.3% underwent SCT. Blood transfusion (71.3%) was the most frequent therapeutic modality (Table 2). Computed tomography (81.3%) and pulmonary function test (52.3%) were the most frequent diagnostic modalities. Patients were admitted with a mean of 3.69 (SD = 2.71) times and a mean duration of each hospital stay of 12.52 (SD = 9.55) days (Table 3).

**Table 3:** Health resource utilization by patients with multiple myeloma.

Health resource utilization in patients with multiple myeloma (N = 227) (per patient) ^†^	Mean	Standard deviation (SD, (Min–Max))
Number of admissions^‡^	3.69	2.71 (1–16)
Average duration of each hospital stay (in days)	12.52	9.55 (1–68.5)
Total duration of hospital stays (in days)	40.25	34.99 (1–247)

^†^Admissions <24 h were excluded from the analysis.

^‡^Number of admissions calculated over the 3-year study period.

**Fig 1:**
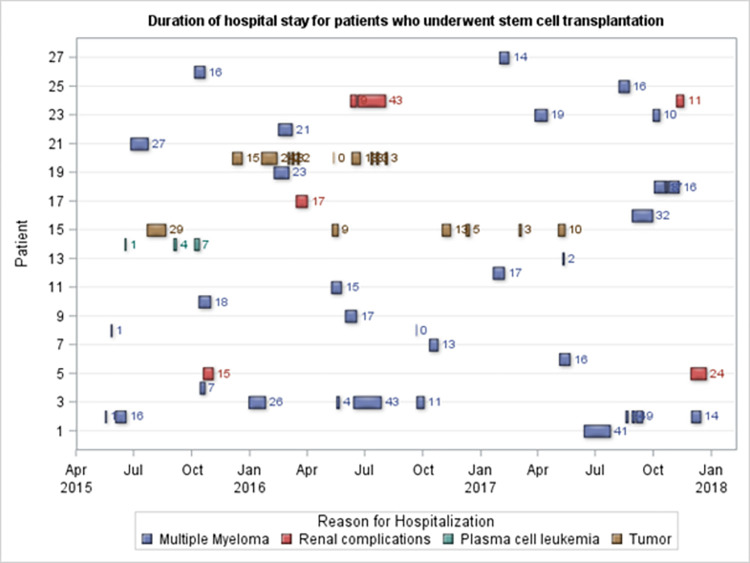
Reason for hospital readmission after SCT. Fig 1 shows the duration of hospital stays (in days) and reason for each admission after a SCT.

**Fig 2:**
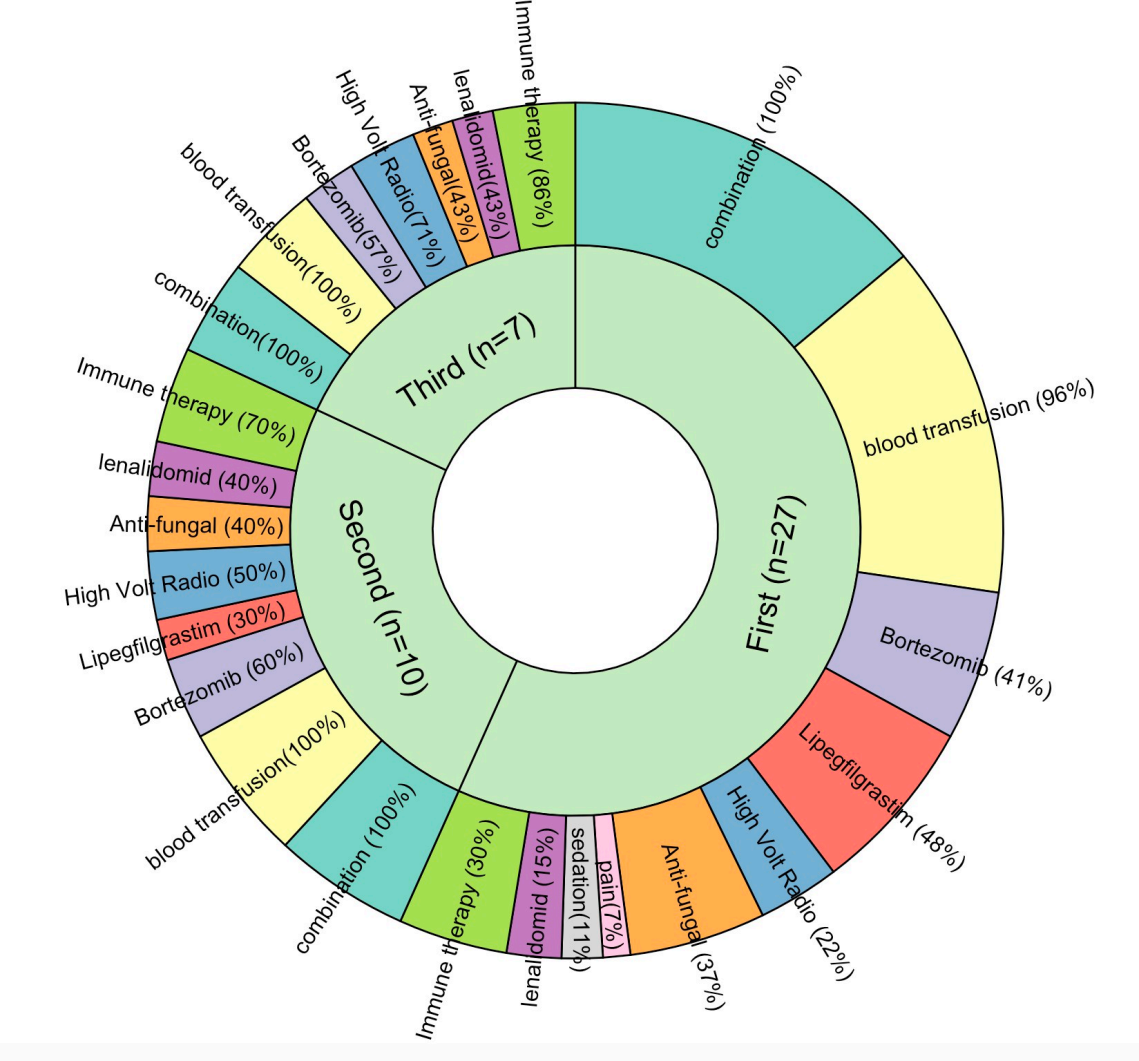
Treatment received during each subsequent admission following stem cell transplantation. Fig 2 shows the treatment provided to SCT patients during the first three readmissions after a SCT procedure.

In the subgroup analysis, procedure date was set as an index date, and patients were followed up prospectively over time. Among patients with SCT (n = 88), 71.6% were aged ≤65 years, with a mean age of 58 years during the first SCT (Table 4). The first SCT was performed after a mean of 98.5 (SD = 83) days from their first recorded admission. Sixty-seven (76.1%) patients underwent a single SCT, and 1 patient received three SCTs during the 3-year study period. Regarding possible disease- and/or treatment-related complications, neutropenia (100%), thrombocytopenia (87.5%), and infection (78.4%) were the most frequent (Table 4). After SCT, 27 (30.7%) patients were readmitted at least once, with MM (56.9%) being the primary reason, followed by other tumors (21.7%; Fig 1). For the first three post-SCT readmissions, combination therapy (100%) and blood transfusions (96.3%–100%) were frequent (Fig 2). Bortezomib (40.7%) and lenalidomide (14.8%) were used post-SCT.

**Table 4:** Characteristics of patients who underwent stem cell transplantation.

SCT-MM patients	N = 88 n (%)
**a. Age distribution**	
≤65years	63 (71.6%)
>65years	25 (28.4%)
Mean age at the time of first stem cell transplantation (SCT; min–max)	58 (34–74)
Number of days from first admission to first SCT, mean (standard deviation)	98.6 (83)
**b. Number of SCT procedures**	
1 SCT	67 (76.1%)
2 SCTs	20 (22.7%)
3 SCTs	1 (1.1%)
**c. Platelet transfusion**	79 (89.77%)
Mean (standard deviation (SD), range)	1.42 (0.63 (1–3))
**d. Treatment-related or disease-related complications in SCT patients**	
Neutropenia	88 (100%)
Thrombocytopenia	77 (87.5%)
Infections	69 (78.4%)
Hypokalemia	56 (63.6%)
Drug-induced anemia	46 (52.3%)

Abbreviations

SCT: Stem cell transplantation

## Discussion

So far very limited information on the usability of German hospital administrative claims data to evaluate patient routine care in complex and rare haematological conditions such as MM has been published. Subsequently, the context of the German federal Medical informatics initiative (MII) and its workstream data integration for future medicine (DiFUTURE) raised the concern on what information the routinely collected data (e.g. the hospital administrative claims data) provides to answer research questions. To our knowledge, the MM use-case provides for the first-time information on inpatient management patterns, resource utilization in a German tertiary teaching hospital. Such information might be used for different purposes like benchmark evaluation and quality assessment.

The hospital administrative claims data contained the data variables that allow the evaluation of demographics, clinical characteristics, management pattern and resource utilization during inpatient stays. Data variables recorded in the used data set are age, gender, ICD– 10 codes to identify comorbidities and treatment-related complications, OPS-code, start date of the procedure, admission and discharge dates, admitting department, reason for admissions, and reason for discharge (transfer to other department/hospital, death, end of treatment course). It was possible to identify MM cases and determine their basic demographic and clinical characteristics. Their treatment patterns and healthcare resource utilization were also evaluated. The availability of information on reimbursed interventions enabled identifying a subgroup of patients who underwent SCT and evaluating their complications and treatment post-SCT from inpatient all-payers perspective. The hospital administrative claims data did not include clinical details such as dates of diagnosis, disease stage, disease severity, response to treatment or laboratory results. Therefore, comprehensive evaluation of MM care from the disease onset was not feasible using the used data set. However, information on disease onset could be drawn from other administrative claims data such as the health insurance databases or patient medical records while clinical details on disease severity, response to treatment and laboratory results are better extracted from patient medical records.

Our study’s basic descriptive results align with previous studies that used administrative claims data in terms of the number of patients with MM and their demographics [1, 2, 22–26], further confirming the availability of data required for case identification and demographic evaluation in the hospital administrative claims dataset. Moreover, the standard of care that involves SCT and administration of novel therapeutic agents such as bortezomib and lenalidomide was integrated into the MM treatment landscape at the KUM. However, the transplantation rate recorded in the hospital administrative claims dataset was higher (38.3% of 230) than that reported by Song et al. [1] (16.2% of 24,507 patients) but consistent with the findings of Rifkin et al. (34% of 1450 patients) [31]. Although we could not construct lines of therapy, we could identify a subset of SCT patients who underwent more than one transplantation. The proportion of patients undergoing a second SCT was slightly higher than that reported by Ashcroft et al. (7% of 337 patients) [32]. A possible explanation for this discrepancy is that our data reflect only on patients with MM hospitalized during the study period because of complications or requiring invasive interventions such as SCT, making them different from patients in outpatient departments or even in other healthcare facilities. Moreover, KUM is a tertiary hospital with a highly specialized hematology–oncology center that receives referrals from other healthcare facilities in Bavaria. Where platelets are considered valuable resources and are not always readily available in the transfusion centers [33], around 90% of our SCT patients received platelet transfusion post-SCT. Such finding indicates the need for a more in-depth evaluation to assess the burden that such an intervention could pose on healthcare facilities and the patients from a health economic perspective.

Although the hospital administrative claims dataset could be used to identify cases and some health-related events, relying on it exclusively for a research study presents several problems. First, its use was primarily restricted to coded health events and interventions during inpatient stays and, therefore, subject to coding comprehensiveness and accuracy which could not be guaranteed and could bias the results. Second, it did not record important data such as the diagnosis date, clinical details, and laboratory and radiological findings, limiting the appropriate retrospective or prospective evaluation of patients from disease onset and the assessment of correlation between health events and disease onset or treatment. Furthermore, the hospital administrative claims dataset did not permit a comprehensive evaluation of patients’ baseline clinical history, disease stage, disease severity, disease progression, and appropriate establishment of the line of therapy due to the unavailability of the aforementioned data. Third, in several instances, ICD-10-GM codes were not precise enough to permit the appropriate evaluation of certain disease conditions or treatment regimens. For example, to identify underweight patients, the ICD-10-GM codes R63.4, abnormal weight loss; R63.6, insufficient intake of food and fluid; and R64, cachexia were used, but none was sufficiently precise to identify underweight cases. The newer version of ICD codes, ICD-11-GM, is more granular and contains a unique code for identifying body mass index-related conditions in adults (ICD-11-GM 5B54, 5B81) [34]. Finally, data required for evaluating health resource utilization, including outpatient and emergency department visits or visits to other departments or even different hospitals, were not recorded. Therefore, the exact disease duration since its first onset could be underestimated, and episodes before the first recorded admission would not be captured. Hence, we could not comprehensively evaluate the patients’ healthcare resource utilization. Moreover, this single center analysis is limited to a single healthcare facility, rendering comparisons with other centers’ datasets or benchmark evaluations impossible. Despite the limited value of the used hospital administrative claims dataset to comprehensively evaluate inpatient management pattern, health resource utilization and quality-of-care in patients with MM, it was able to provide some insight that require future comprehensive analysis. By contrast, single-payer claims data (e.g., SHI) provide data variables that complement those from hospital administrative claims data (e.g., disease onset, health provisions from outpatients and emergency department visits and cross-sector information). However, German administrative claims data, in general, lack clinical details on disease stage/severity, response to treatment, laboratory results, and possibility to distinguish between disease-related and/or treatment-related complications. Therefore, researchers aiming at addressing any of these aspects will have to supplement data by utilizing other data sources such as patient medical records, pharmacy records, laboratory files and health insurance databases for intersectoral analyses.

Our observations of the limitations of the hospital administrative claims dataset in terms of adequately evaluating clinical characteristics and disease progression agree with previous reports [5, 15, 18]. However, the recorded information in our dataset on therapeutics and diagnostic procedures provided to patients during hospitalization allowed us to evaluate the treatment pattern in the SCT group. Similarly, Kreis K. et al. reported the limitations of German claims data to evaluate treatment patterns and assess treatment discontinuation/switching due to missing clinical details [14]. Our results are also consistent with previous studies on the limitations of administrative claims data in terms of adequately evaluating the incidence of adverse events [29–31]. However, the hospital administrative claims dataset provided some quality indicators, e.g., infections, readmissions, and platelet transfusion rates among SCT patients, signaling possible adverse events that require further evaluation. Such indicators are essential for evaluating the economic impact of the disease. Fonseca et al. reported that multiple admissions and treatment- or disease-related complications have some effect on disease financial burden [2]. They are also crucial for healthcare management evaluation within a healthcare facility overtime or for benchmark evaluations, which compare the quality of care among different healthcare facilities, such as guideline adherence and complications post treatment [35, 36]. One of the objectives of the BMBF and MII is to support the use of routine-care data in health research and the exchange of data between different German healthcare institutions [37]. Thus, allowing for comprehensive quality-of-care assessments and benchmark evaluation between different healthcare facilities in Germany. We believe that the dataset can be used for quality indicator and guideline adherence assessment either within the hospital or compared with other tertiary hospitals sharing a similar database infrastructure. Future research should consider linking claims datasets to other secondary data sources to rigorously evaluate disease characteristics, treatment patterns, treatment-related adverse events, and economic burden of MM management from broader perspectives. It must also consider evaluating the quality of care concerning complications and number of admissions after a medical intervention or a novel therapy to enable a more in-depth evaluation of treatment effectiveness.

## Conclusions

German hospital administrative claims data are an important information source to identify cases, medical events, and outcomes of interest based on predefined criteria in rare conditions such as MM from inpatient settings. Patients with MM identified from the dataset had complications such as infections, which indicate the need for more in-depth evaluations for quality-of-care assessment and benchmark evaluation compared with other healthcare facilities. Furthermore, key elements such as complications, treatment frequency, and readmission rates were available in the dataset, rendering it a useful secondary data source for health service research studies. However, a comprehensive evaluation from both inpatient and outpatient settings of clinical characteristics, management pattern, healthcare resource utilization, and quality of care of patients with MM requires linking hospital administrative claims data to other secondary data sources.

## Supporting information

S1 Table Data elements required to answer questions related to multiple myeloma. (DOCX)

## References

[1] Song X, Cong Z, Wilson K. Real-world treatment patterns, comorbidities, and disease-related complications in patients with multiple myeloma in the United States. Curr Med Res Opin. 2016;32(1):95–103. doi: 10.1185/03007995.2015.1105202 26488820

[2] Fonseca R, Abouzaid S, Bonafede M, et al. Trends in overall survival and costs of multiple myeloma, 2000–2014. Leukemia. 2017;31(9):1915–21. doi: 10.1038/leu.2016.380 28008176 PMC5596206

[3] Teitelbaum A, Ba-Mancini A, Huang H, et al. Health Care Costs and Resource Utilization, Including Patient Burden, Associated With Novel-Agent-Based Treatment Versus Other Therapies for Multiple Myeloma: Findings Using Real-World Claims Data. Oncologist. 2013; doi: 10.1634/theoncologist.2012-0113 23299776 PMC3556254

[4] Heller G. Administrative Data from Germany’s Statutory Health Insurances for Social, Economic and Medical Research. German Council for Social and Economic Data (RatSWD). 2009.

[5] Neubauer S, Kreis K, Klora M, et al. Access, use, and challenges of claims data analyses in Germany. Eur J Heal Econ. 2017;18(5):533–6. 10.1007/s10198-016-0849-3 27878393

[6] McGuigan A, Kelly P, Turkington RC, Jones C, Coleman HG, McCain RS. Pancreatic cancer: A review of clinical diagnosis, epidemiology, treatment and outcomes. World J Gastroenterol. 2018;24(43):4846–61. doi: 10.3748/wjg.v24.i43.4846 30487695 PMC6250924

[7] Birnbaum HG, Cremieux PY, Greenberg PE, et al. Using Healthcare Claims Data for Outcomes Research and Pharmacoeconomic Analyses CURRENT OPINION. Pharmacoeconomics. 1999;16(1):1–8. doi: 10.2165/00019053-199916010-00001 10539118

[8] Brandes A, Schwarzkopf L, Rogowski WH. Using claims data for evidence generation in managed entry agreements. Int J Technol Assess Health Care. 2016;32(1):69–77. doi: 10.1017/S0266462316000131 26975757

[9] Gansen FM. Health economic evaluations based on routine data in Germany: a systematic review. BMC Health Serv Res [Internet]. 2018 Dec 10;18(1):268. Available from: https://bmchealthservres.biomedcentral.com/articles/10.1186/s12913-018-3080-3 29636046 10.1186/s12913-018-3080-3 PMC5894241

[10] Majhail NS, Mau LW, Denzen EM, et al. Costs of autologous and allogeneic hematopoietic cell transplantation in the United States: a study using a large National Private Claims Database. Bone Marrow Transplant. 2012;48:294–300. doi: 10.1038/bmt.2012.133 22773126 PMC3469749

[11] Preussler JM, Mau L-W, Majhail NS, et al. Administrative Claims Data for Economic Analyses in Hematopoietic Cell Transplantation: Challenges and Opportunities. Biol Blood Marrow Transplant. 2016;22:1738–46. doi: 10.1016/j.bbmt.2016.05.005 27184624 PMC5600540

[12] Schwarzkopf L, Wacker M, Holle R, et al. Cost-components of lung cancer care within the first three years after initial diagnosis in context of different treatment regimens. Lung Cancer. 2015;90:274–80. doi: 10.1016/j.lungcan.2015.09.005 26384433

[13] Datenlieferung gem. § 21 KHEntgG, InEK GmbH [Internet]. [cited 2020 Feb 13]. Available from: https://www.g-drg.de/Datenlieferung_gem._21_KHEntgG

[14] Kreis K, Neubauer S, Klora M, et al. Status and perspectives of claims data analyses in Germany-A systematic review. Health Policy (New York). 2016;120(2):213–26. doi: 10.1016/j.healthpol.2016.01.007 26826756

[15] Scheid C, Blau IW, Sellner L, Ratsch BA, Basic E. Changes in treatment landscape of relapsed or refractory multiple myeloma and their association with mortality: Insights from German claims database. Eur J Haematol. 2020;106:148–57. doi: 10.1111/ejh.13523 32989806 PMC7894176

[16] Ohlmeier C, Saum KU, Galetzka W, Beier D, Gothe H. Epidemiology and health care utilization of patients suffering from Huntington’s disease in Germany: Real world evidence based on German claims data. BMC Neurol. 2019;19(1):1–8. 31823737 10.1186/s12883-019-1556-3 PMC6905058

[17] Healthcare Financial Management Association. Costing healthcare in Germany. 2015; Available from: www.hfma.org.uk

[18] Elixhauser A, Steiner C, Harris DR, et al. Comorbidity Measures for Use with Administrative Data. Med Care. 1998;36(1):8–27. doi: 10.1097/00005650-199801000-00004 9431328

[19] Maier B, Wagner K, Behrens S, Bruch L, Busse R, Schmidt D, et al. Comparing routine administrative data with registry data for assessing quality of hospital care in patients with myocardial infarction using deterministic record linkage. BMC Health Serv Res [Internet]. 2016 Oct 21 [cited 2022 Feb 23];16(1):1–9. Available from: https://bmchealthservres.biomedcentral.com/articles/10.1186/s12913-016-1840-5 27769288 10.1186/s12913-016-1840-5 PMC5073420

[20] Moreau P, San Miguel J, Sonneveld P, et al. Multiple myeloma: ESMO Clinical Practice Guidelines for diagnosis, treatment and follow-up. Ann Oncol. 2017;28:iv52–61. doi: 10.1093/annonc/mdx096 28453614

[21] Turesson I, Velez R, Kristinsson SY, Al E. Patterns of multiple myeloma during the past 5 decades: Stable incidence rates for all age groups in the population but rapidly changing age distribution in the clinic. Mayo Clin Proc. 2010;85(3):225–30. doi: 10.4065/mcp.2009.0426 20194150 PMC2843108

[22] Gerecke C, Fuhrmann S, Strifler S, et al. The Diagnosis and Treatment of Multiple Myeloma. Dtsch Aerzteblatt Online. 2016;(1):470–7. doi: 10.3238/arztebl.2016.0470 27476706 PMC4973001

[23] Pulte D, Jansen L, Castro FA, Al. E. Trends in survival of multiple myeloma patients in Germany and the United States in the first decade of the 21st century. Br J Haematol. 2015;171(2):189–96. doi: 10.1111/bjh.13537 26123295

[24] Denz U, Haas PS, Wä Sch R, et al. State of the art therapy in multiple myeloma and future perspectives. Eur J Cancer. 2006;42(11):1591–600. doi: 10.1016/j.ejca.2005.11.040 16815703

[25] Koleva D, Cortelazzo S, Toldo C, et al. Healthcare costs of multiple myeloma: An Italian study. Eur J Cancer Care (Engl). 2011;20(3):330–6. 20148933 10.1111/j.1365-2354.2009.01153.x

[26] Moehler TM, Merz M, Kellermann L, Goldschmidt H, Knauf W. Diagnostic and therapeutic approaches to multiple myeloma patients: ‘Real-world’ data from representative multicentre treatment surveys in Germany between 2008 and 2011. Oncol Lett. 2016;12(6):5043–51. doi: 10.3892/ol.2016.5375 28105211 PMC5228488

[27] Merz M, Kellermann L, Poenisch W, Al E. Diagnosis and treatment of multiple myeloma in Germany: analysis of a nationwide multi-institutional survey. Ann Hematol. 2017;96(6):987–93. doi: 10.1007/s00277-017-2991-0 28409228

[28] Yong K, Delforge M, Driessen C, et al. Multiple myeloma: patient outcomes in real-world practice. Br J Haematol. 2016;175(2):252–64. doi: 10.1111/bjh.14213 27411022 PMC5096152

[29] Benchimol EI, Smeeth L, Guttmann A, Harron K, Moher D, Peteresen I, et al. The REporting of studies Conducted using Observational Routinely-collected health Data (RECORD) Statement. PLoS Med. 2015;12(10):1–22. 10.1371/journal.pmed.1001885 PMC4595218 26440803

[30] Palmaro A, Gauthier M, Despas F, Lapeyre-Mestre M. Identifying cancer drug regimens in French health insurance database: An application in multiple myeloma patients. Pharmacoepidemiol Drug Saf. 2017;26(12):1492–9. doi: 10.1002/pds.4266 28745019

[31] Rifkin RM, Jagannath S, Durie BGM, et al. Treatment Outcomes and Health Care Resource Utilization in Patients With Newly Diagnosed Multiple Myeloma Receiving Lenalidomide-only Maintenance, Any Maintenance, or No Maintenance: Results from the Connect MM Registry. Clin Ther. 2018;40(7):1193–1202.e1. doi: 10.1016/j.clinthera.2018.05.017 30007443

[32] Ashcroft J, Judge D, Dhanasiri S, et al. Chart review across EU5 in MM post-ASCT patients. Int J Hematol Oncol. 2018;7(1):IJH05. doi: 10.2217/ijh-2018-0004 30302236 PMC6176952

[33] Alcaina PS. Platelet transfusion: And update on challenges and outcomes. J Blood Med. 2020;11:19–26. doi: 10.2147/JBM.S234374 32158298 PMC6986537

[34] ICD-11—Mortality and Morbidity Statistics [Internet]. [cited 2019 Dec 7]. Available from: https://icd.who.int/browse11/l-m/en#/http%3A%2F%2Fid.who.int%2Ficd%2Fentity%2F1153296343

[35] Maise EM, Evans KA, Chu B-C, et al. Temporal Trends in Survival and Healthcare Costs in Patients with Multiple Myeloma in the United States. Am Heal Drug Benefit. 2018;11(1):39–46. PMC5902764 29692879

[36] Lovaglio PG. Benchmarking strategies for measuring the quality of healthcare: Problems and prospects. Sci World J. 2012;2012:606154. doi: 10.1100/2012/606154 22666140 PMC3361319

[37] Semler SC, Wissing F, Heyder R. German Medical Informatics Initiative. Methods Inf Med. 2018 Jul 1;57(S 01):e50–6. doi: 10.3414/ME18-03-0003 30016818 PMC6178199

