# Supplementary material for: Usability of German hospital administrative claims data for healthcare research: General assessment and use case of multiple myeloma in Munich university hospital in 2015–2017
Source: PLoS One. 2022 Jul 28;17(7):e0271754. doi: 10.1371/journal.pone.0271754 (PMC9333282; doi:10.1371/journal.pone.0271754)
Supplement: S1 Table — (DOCX) [file pone.0271754.s001.docx]

**S1 Table:**

**Table:** Data elements required to answer questions related to multiple myeloma.

| **Aspect** | **Question** | **Required variables** | **Additional details (Tests or ICD/OPS code)** | **Possible source of information** |
| --- | --- | --- | --- | --- |
| **Demographics** | 1. How can the patients be identified? | Patient’s unique identifier | - | PMC  EHR  CD |
|  | 2. What is the age distribution for the identified group of patients? | Age | Date of birth | PMC  EHR  CD |
|  | 3. What is the sex distribution of the disease? | Sex | - | PMC  EHR  CD |
|  | 4. In which departments were the patients admitted? | Admitting department | -  -  - | PMC  EHR  CD |
| **Epidemiology and Clinical characteristics** | 1. How can multiple myeloma be identified? | I. Documented diagnosis of multiple myeloma | C90.00  C90.01  C90.00+  C90.01+ | PMC  EHR  CD |
|  | 2. How the diagnosis of multiple myeloma be confirmed? (other possible criteria needed for identifying the disease) | I. Laboratory tests | 1. Serum or urinary protein electrophoresis (8-82).  2. Nephelometric quantification of immunoglobulins.  3. Immunofixation.  4. Bone marrow biopsies/aspiration for measurement of plasma level (1-424, 1-941).  5. Serum FLC level.  6. Complete blood count with differential serum creatinine, creatinine clearance, and calcium level. | PMC  EHR |
|  |  | II. Radiologic tests | 1. WBLD-CT (3-20 – 3-26)  2. MRI (3-80 – 3-84)  3. PET-CT (3-75)  4. Conventional radiographs. | PMC  HER |
|  | 3. How can disease stage of multiple myeloma patients be assessed? | I. International staging system (ISS) for multiple myeloma. | 1. Serum β2M level.  2. Serum Albumin. | PMC  EHR |
|  | 4. How can risk be assessed in the identified group of patients? | I. ISS  II. Chromosomal abnormality detection (iFISH).  III. LDH level. | Cytogenetic testing (1-991 – 1-999) | PMC  EHR |
|  | 5. What are the most common comorbid conditions present in this disease group? | **Concurrent comorbidities:** |  | PMC  EHR  ?CD |
|  |  | Hypertension | I10 – I15 |  |
|  |  | Congestive heart failure | I50 |  |
|  |  | Cerebrovascular disease | I60 – I69 |  |
|  |  | Peripheral vascular disease | I70 – I79 |  |
|  |  | Myocardial infarction | I20 – I24 |  |
|  |  | Chronic kidney disease | N18 |  |
|  |  | Primary tumor | C00 – C97  D00 – D48 |  |
|  | 6. What are the most commonly reported disease-related and/or treatment-related complications in the identified disease group? | **Disease or treatment complications:** |  | PMC  EHR |
|  |  | Bone lesions | M82.0 |  |
|  |  | Multiple myeloma-related renal disease | N16.1* |  |
|  |  | Multiple myeloma-related glomerular disease | N08.1* |  |
|  |  | Neoplasm-induced anemia | D63.0* |  |
|  |  | Thrombocytopenia | D69.5, D69.6 |  |
|  |  | Neutropenia | D70. |  |
|  |  | Hypercalcemia | E83.5 |  |
|  |  | Thromboembolic event | I82 |  |
|  |  | Gastrointestinal bleeding | K92. |  |
|  |  | Cerebral hemorrhage | I60. – I62. |  |
|  |  | Treatment complication | Y57, Y69, Y84 |  |
|  |  | Death | R96, R98, R99 | PMC  EHR  CD |
|  |  |  |  |  |
| **Treatment pattern** | 1. What medications are used as a front-line therapy in the disease group? | Bortezomib | 6-001.9 | PMC  EHR  CD(-)  Pharmacy files |
|  |  | Melphalan | NA |  |
|  |  | Prednisone | NA |  |
|  |  | Dexamethasone | NA |  |
|  |  | Cyclophosphamide | NA |  |
|  |  | Carfilzomib | 6-008.9 |  |
|  |  | Thalidomide | NA |  |
|  |  | Lenalidomide | 6-003.g |  |
|  |  | Pomalidomide | 6-007.a |  |
|  |  | Combination chemotherapy | 8-542  8-543  8-544  8-547 |  |
|  |  | Panobinostat | 6-009.2 |  |
|  |  | Elotuzumab | 6-009.d |  |
|  |  | Ixazomib | NA |  |
|  |  | Daratumumab | 6-009.a |  |
|  |  | Stem cell transplantation | 5-410  5-411  8-860 |  |
|  |  | Transfusion of hematopoietic stem cell | 8-805 |  |
|  | 2. What medications are used to treat relapsed/refractory multiple myeloma in the identified group? | Medications listed above |  | PMC  EHR  CD(-)  Pharmacy files |
|  | 3. What medications are used for consolidation therapy in the identified group? | Medications listed above |  | PMC  EHR  CD(-)  Pharmacy files |
|  | 4. What medications are used for maintenance therapy in the identified disease group? | Medications listed above |  | PMC  EHR  CD(-)  Pharmacy files |
|  | 5. What medications are used for supportive care in the disease group? | Anti-fungal medications | 6-002.5  6-002.p  6-002.q  6-002.r  6-003.1  6-004.5 | PMC  EHR  CD(-)  Pharmacy files |
|  |  | Blood product transfusion | 8-800 |  |
|  |  | Thrombocyte transfusion | 8-800.g |  |
|  |  | Hemodialysis | 8-854 |  |
|  |  | Therapeutic plasmapheresis | 8-820 |  |
|  |  | Sedation | 8-903 |  |
|  |  | Lipegfilgrastim | 6-007.7 |  |
|  |  | High voltage radiotherapy | 8-522 |  |
|  |  | Pain therapy | 8-91 |  |
|  | Further required information: | Start date of treatment | - | PMC  EHR  Pharmacy files |
|  |  | End date of treatment | - |  |
|  |  | Dose of medication | - |  |
|  |  | Route of administration | - |  |
| **Health service utilization** | 1. How frequently were multiple myeloma patients admitted to the hospital? | Date of admission |  | PMC  EHR  CD |
|  | 2. How long (on average) did multiple myeloma patients stay at the hospital? | Date of discharge |  | PMC  EHR  CD |
|  | 3. To which hospital departments were the identified group of patients mostly admitted? | Admitting department |  | PMC  EHR  CD |
|  | 4. What health services were mostly used by the identified group of patients? | I. Laboratory  II. Radiological  III. Pharmacy  IV. ICU  V. Operation |  | PMC  EHR  CD |
|  |  |  |  |  |
|  | Further required information: | Reason for admission reason |  | PCD  EHR |
|  |  | Reason for discharge |  | PMC  EHR |
| PMC = Patient medical chart; EHR = Electronic hospital record; CD=Claims data; FLC= Free-light chain measurement.; WBLD-CT= Whole body low dose-computed tomography; MRI = Magnetic resonance Imaging; PET-CT =Positron emission tomography with CT; β2M = β2 microglobulin; LDH=Lactate-dehydrogenase; iFISH= inter-phase fluorescent in situ hybridization; NA = not available; ICU: Intensive care unit; ICD = International Classification of Diseases; OPS = Procedure codes | | | | |
